# Clearance Kinetics and Matrix Binding Partners of the Receptor for Advanced Glycation End Products

**DOI:** 10.1371/journal.pone.0088259

**Published:** 2014-03-18

**Authors:** Pavle S. Milutinovic, Judson M. Englert, Lauren T. Crum, Neale S. Mason, Lasse Ramsgaard, Jan J. Enghild, Louis J. Sparvero, Michael T. Lotze, Tim D. Oury

**Affiliations:** 1 Department of Pathology, University of Pittsburgh School of Medicine, Pittsburgh, Pennsylvania, United States of America; 2 Department of Medicine, Brigham and Women's Hospital, Boston, Massachusetts, United States of America; 3 Department of Radiology, University of Pittsburgh School of Medicine, Pittsburgh, Pennsylvania, United States of America; 4 Center for Insoluble Protein Structures, Department of Molecular Biology and Genetics, Aarhus University, Aarhus, Denmark; 5 Department of Surgery, University of Pittsburgh Cancer Institute, Pittsburgh, Pennsylvania, United States of America; New York University, United States of America

## Abstract

Elucidating the sites and mechanisms of sRAGE action in the healthy state is vital to better understand the biological importance of the receptor for advanced glycation end products (RAGE). Previous studies in animal models of disease have demonstrated that exogenous sRAGE has an anti-inflammatory effect, which has been reasoned to arise from sequestration of pro-inflammatory ligands away from membrane-bound RAGE isoforms. We show here that sRAGE exhibits *in vitro* binding with high affinity and reversibly to extracellular matrix components collagen I, collagen IV, and laminin. Soluble RAGE administered intratracheally, intravenously, or intraperitoneally, does not distribute in a specific fashion to any healthy mouse tissue, suggesting against the existence of accessible sRAGE sinks and receptors in the healthy mouse. Intratracheal administration is the only effective means of delivering exogenous sRAGE to the lung, the organ in which RAGE is most highly expressed; clearance of sRAGE from lung does not differ appreciably from that of albumin.

## Introduction

Advanced glycation end products (AGEs) are proteins or lipids with sugar moieties incorporated via non-enzymatic organic reactions, such as the Maillard reaction. Normally generated endogenously at low levels, AGE formation is greatly amplified in the hyperglycemic and oxidative state in diabetes. Although at the time of RAGE's discovery other proteins were known to mediate AGE uptake by phagocytic cells [1], [2], it was only with the discovery of endothelial expression of RAGE that an elegant mechanism relating elevated AGEs to vascular complications of diabetes was in place [3], [4]. Research since then has shown RAGE to be an immunoglobulin superfamily receptor which binds and signals in response to a vast array of endogenous mediators, including amphoterin (HMGB1) [5], [6], amyloid beta [7], calgranulins (S100 proteins) [8], [9], [10], and the integrin CD11b (Mac-1) [11], [12]. It is therefore not surprising that this protein is now known to be an important player in a number of disease processes in humans and animal models, including cardiomyopathy [8], neurodegeneration [13], and tumor invasion [14]. The rich body of literature establishing RAGE's role as a pattern recognition receptor in a broad spectrum of inflammatory disease processes notwithstanding, its function in the healthy state remains nebulous.

RAGE exists as two major isoforms: a membrane form of RAGE, thought to drive downstream inflammatory cascades, and its soluble counterpart, sRAGE, which has been shown in most disease models to have a contravening, salutary effect: sRAGE acts as a decoy receptor by sequestering ligands away from the membrane isoform, thereby inhibiting inflammation. Substantial evidence has been presented in support of this model of RAGE biology [15], [16], [17], [18]. Expression profiling has demonstrated that RAGE is expressed in the healthy lung at overwhelmingly greater levels than in any other tissue assayed [12], [19], [20]. Furthermore, numerous studies have shown that pulmonary RAGE is primarily expressed on the basolateral surface of type I alveolar epithelial cells [21], [22], [23]. This would suggest that one or both isoforms of RAGE have an important function in pulmonary homeostasis, most likely as a sensor of environmental cues or as a mediator of cell adhesion to underlying basement membrane. Indeed, RAGE's affinity for heparin (which is exploited in its purification), as well as the observation made by other investigators of specific binding by RAGE-expressing cells to ECM-coated surfaces [24], indicates that there is already substantial evidence for RAGE binding to ECM components. Whether RAGE binds to ECM directly or via intermediary macromolecules is unknown, as is the identity of the particular ECM proteins that interact with RAGE.

Soluble RAGE has been administered as a therapeutic agent in a number of animal models of disease and has in most cases been shown to have a beneficial effect [25], [26], [27], [28], [29], [30], [31], [32]. While this is usually explained through allusion to a decoy mechanism, which has garnered much supportive evidence in the form of *in vitro* competitive inhibition studies [33], sRAGE's anti-inflammatory effects could also arise from ligation of an as yet unidentified receptor. Furthermore, the affinity of RAGE to ECM [24] suggests that the latter could serve as a sink for endogenously-shed or exogenously-administered sRAGE, in much the same way that ECM retains growth factors such as FGF. To address these hypotheses, as well as answer the more practical question of which delivery method would best exploit sRAGE's therapeutic potential in models of lung disease, radiolabeled sRAGE clearance studies were undertaken to probe for sRAGE binding partners and sites of distribution *in vivo*.

## Experimental Procedures

### Ethics statement

Animal experiments were performed in accordance with a protocol (protocol number: 1004847) reviewed and approved by the Institutional Animal Care and Use Committee at the University of Pittsburgh. All effort was made to minimize suffering during treatments, and surgery was performed following anesthetization with sodium pentobarbital. Human lung tissue used in preparing purified proteins, including sRAGE, was obtained from autopsies performed at the University of Pittsburgh. Approval for this was obtained from the University of Pittsburgh Committee for Oversight of Research and Clinical Training Involving Decedents (CORID). All autopsy consent forms at the University include a statement that indicates that autopsy tissue may be used for research, and the Committee has indicated that additional consent is not required and that our use of such tissue for protein purification is approved (CORID No. 266).

### sRAGE purification from lung, endotoxin removal, concentration and biologic activity determination

Soluble RAGE was purified from mouse and human lungs according to methods previously described [34], [35]. Soluble RAGE was rendered free of detectable endotoxin (<5 EU/kg mouse) using Detoxi-Gel polymyxin B resin (Thermo Scientific) per manufacturer's instructions. Mouse sRAGE concentration was determined using the molar extinction coefficient and absorbance at 280 nm (see below). Following purification of mouse sRAGE, biologic activity was tested and confirmed using HMGB1 concentration-dependent binding as a marker of molecular integrity, according to previously described methods [19], [36] (data not shown).

### sRAGE molar extinction coefficient determination

The concentration of mouse or human RAGE was determined by quantitative amino acid analysis. The analyses were performed in triplicate with approximately 2 µg of purified mouse or human soluble RAGE. The samples were dried in 500-µl polypropylene vials, the lids were punctured and the vials placed in a 25-ml glass vial equipped with a MinInert valve (Pierce Biotechnology). A total of 200 µl 6 N HCl containing 0.1% phenol was placed in the bottom and the glass was purged with argon before vacuum was applied. The samples were incubated at 110°C for 18 h. The samples were subsequently re-dissolved in 50 µl of 0.2 M sodium citrate loading buffer at pH 2.2 (Biochrom, Cambridge, UK), transferred into microvials and loaded onto a BioChrom 30 amino acid analyzer (Biochrom). Data analysis was performed using in-house developed software. The extinction coefficients were calculated based on the determined protein concentrations and the UV absorbance at 280 nm.

### Biolayer interferometry

Biolayer interferometry (BLI) was performed on a ForteBIO Octet (Menlo Park, CA) to determine the kinetics of RAGE binding to collagen I, collagen IV, laminin, and fibronectin (all from Sigma) per manufacturer's instructions and published methods [37], [38]. Purified mouse soluble RAGE was conjugated to amine-reactive sensor tips. The tips were then transferred into wells containing the matrix protein of interest in varying concentrations. Binding was measured as a deflection in the wavelength of light passing through the sensor. Following 1800 s of incubation, the RAGE-coated sensor tips were then transferred to PBS to allow dissociation. In order to confirm specificity of the binding, reciprocal binding studies were performed with extracellular matrix proteins coupled to the sensor, with sRAGE in solution. Binding curves were analyzed using the ForteBIO software, which performs a global fit according to the 1∶1 Langmuir model in order to obtain the kinetic rate constants for each set of interaction conditions [37].

### Radiolabeling of mouse serum albumin and mouse sRAGE

Chromatographically purified mouse serum albumin (Sigma) and mouse sRAGE were labeled with Na^125^I (Perkin Elmer) using the chloramine T method (Iodination Beads, Pierce) per manufacturer's instructions. To remove residual iodine, both samples were run over Bio-Spin 6 (Bio-Rad) centrifugation columns and the eluate was collected. Protein concentration was determined using the absorbance at a wavelength of 280 nm, the calculated molar extinction coefficient of mouse sRAGE (see Results), and the commonly reported mass extinction coefficient of serum albumin of 0.52×10^−3^ mL µg^−1^ cm^−1^. Gamma counting was performed on a Cobra II auto gamma counter (Packard, Perkin-Elmer) and activity calculated from disintegrations per minute and counting efficiency. Samples were analyzed by SDS-PAGE, Coomassie Blue staining, and autoradiography to assess purity.

### Animal studies

All animal experiments were approved by the institutional animal care and use committee. Eight-week-old male C57BL/6 mice (Charles River) were administered radioiodinated mouse serum albumin or mouse sRAGE via intratracheal (i.t.), intraperitoneal (i.p.), or intravenous (i.v.) routes. Each animal received 1 µCi of radiolabeled protein in saline, which corresponded to ∼0.6–0.9 µg of mouse serum albumin or ∼0.7–1.4 µg of mouse sRAGE; for intraperitoneal/intravenous and intratracheal treatments, the treatment volumes were 200 µL and 70 µL, respectively. Mice were sacrificed at 1, 2, 4, and 12 hours after the treatment, with 4–5 mice used per treatment group (the variables being test vs. control, route of administration, and time of sacrifice post-treatment). Mice were euthanized with i.p. sodium pentobarbital, urine was collected, and blood drawn from the right ventricle of the heart. The pulmonary circulation was transcardially perfused with saline until the lungs blanched. Then, the systemic circulation was transcardially perfused with saline until the liver and kidneys blanched. In 3 mice of each group, the lungs were inflation-fixed with 800 µL of 10% neutral buffered formalin (NBF) and the systemic circulation was perfused with 10% NBF, as well. Following organ harvest, the stomach, small intestine, and colon were thoroughly flushed with saline before weighing and gamma counting.

### Tissue, fluid, and organ processing

The following tissues, fluids, and organs were assayed: blood, urine, stomach, small intestine, colon, bladder, kidneys, pancreas, spleen, liver, skeletal muscle, femur, thymus, heart, lungs, and brain. These were dispensed into previously tared gamma counting vials and weighed. Samples were kept on ice until ready for counting. Following gamma counting, unfixed samples were transferred to cryotubes and flash frozen in liquid nitrogen and stored at −80°C; fixed organs, excepting bone, were placed in cassettes and agitated in a large volume of 10% NBF overnight; bones were decalcified in Cal-Rite (Richard Allen Scientific) overnight. Following fixation, samples were dehydrated through an ethanol series, pre-cleared in xylenes, and embedded in paraffin.

### SDS-PAGE and autoradiography of organ homogenates

Frozen lungs, kidneys, livers, stomachs, and pancreata of mice that were treated i.t., i.p., or i.v. with radiolabeled mouse serum albumin or mouse sRAGE were homogenized in 1.5 mL ice-cold homogenization buffer (50 mM Tris-HCl, 150 mM NaCl, pH 7.4) containing protease inhibitors (100 µM DCI, 1 mM 1,10-phenanthroline, and 10 µM E-64). The homogenates were centrifuged at 20000× *g* for 20 minutes at 4°C. Supernatants were collected and 60 µL of each sample was denatured and reduced with SDS and DTT and boiled for 3 minutes, then shock cooled on ice. Proteins were separated by SDS-PAGE and detected by Coomassie Blue staining and autoradiography to assess radiolabeled protein degradation as a function of time.

### Slide preparation and autoradiographic analysis

Paraffin blocks were sectioned at a thickness of 5 µm and tissue sections were mounted on glass slides and air-dried. Deparaffinization was accomplished with heat and xylenes, and the tissue was rehydrated through an ethanol series. Autoradiography emulsion NBT (Eastman Kodak, Rochester, NY) was heated to 42°C. Slides were dipped in molten emulsion, briefly air dried, and stored in light-insulated slide boxes with desiccant. Slides were stored at 4°C in the dark for 3–5 weeks. Following incubation, the slides were developed in D-19 developer and fixed (developer and fixer both from Eastman Kodak), then washed, stained with hematoxylin and eosin, dehydrated into xylenes, and coverslipped with Permount (Fisher). Slides were examined using an Olympus BH-2 microscope equipped with an Olympus DP12 camera.

### Regression and statistical analysis

Quantitative data were analyzed using GraphPad Prism 5 (GraphPad Software Inc., La Jolla, CA). Exponential decay fitting for radioactive tracer clearance was performed using this program, as were statistical analyses. Comparisons were conducted with Student's t-test. Values are reported as mean ± standard error of the mean. A *p*-value of <0.05 was considered statistically significant and is indicated by an asterisk on graphical representations of the data.

## Results

### Determination of sRAGE molar extinction coefficient

Because sRAGE is widely used as a therapeutic in animal models of disease, and in view of numerous studies that have aimed to quantitatively characterize sRAGE interactions with ligands, the molar extinction coefficients of mouse and human sRAGE (in both cases with the 21–22 residue signal peptide cleaved off) at 280 nm light wavelength and 22°C, were determined. Due to interspecies variation in glycosylation and intrinsic characteristics unique to the protein, these coefficients are expected to permit more accurate quantitation than was possible based on colorimetric absorbance-shift methods. Table 1 summarizes the results, which demonstrate a marked discrepancy from the theoretically predicted molar extinction coefficients. This is most likely related to the contribution of carbohydrate moieties to the measured absorbances of mouse and human sRAGE.

**Table 1:** Mouse and human sRAGE extinction coefficients.

Description	Amino acid residues	Mass (*Da*)	Extinction coefficient (*M^−1^ cm^−1^*)	Theoretically predicted^c^ extinction coefficient (*M^−1^ cm^−1^*)
Mouse sRAGE	22–330^a^	35742.30	67573	33835
Human sRAGE	23–347^b^	37939.72	87237	39335

a Mouse membrane RAGE has a published sequence length of 402 (NP_031451.2).

b Human membrane RAGE has a published sequence length of 404 (NP_001127.1).

c [50].

### Determination of sRAGE affinities for extracellular matrix proteins

**Figure 1:**
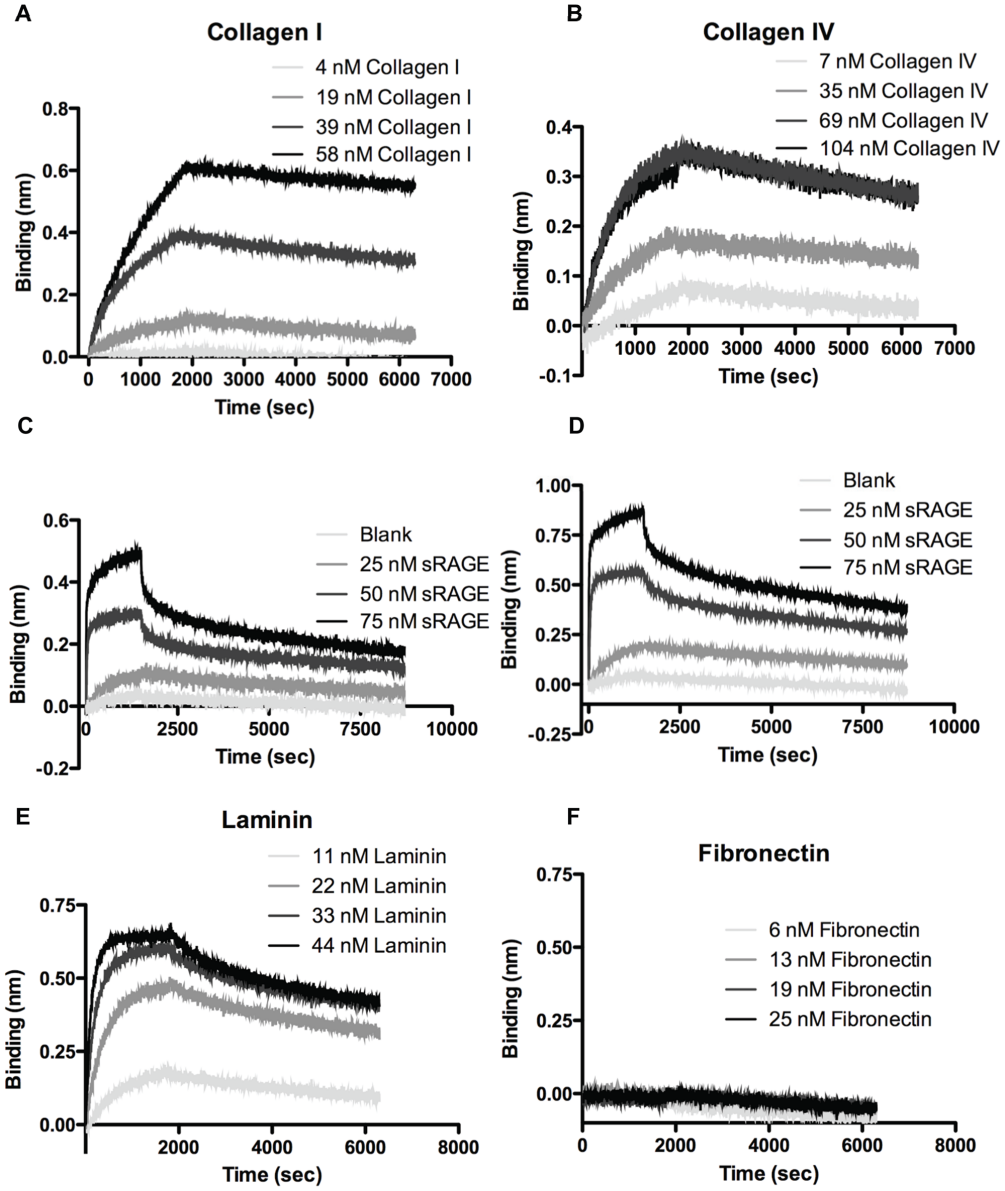
RAGE binds with high affinity to collagen I, collagen IV, and laminin, but not to fibronectin. Soluble RAGE affinity for collagen I, collagen IV, laminin, and fibronectin was assessed by biolayer interferometry. Soluble RAGE was conjugated to an amine-reactive sensor and dipped into solutions containing collagen I (A), collagen IV (B), laminin (E), or fibronectin (F) for 1800 s, at which point dissociation was initiated by washing in PBS. Binding was measured as a deflection in the wavelength of light passing through the sensor; affinities were calculated by fitting the curves with analysis software. Reciprocal binding was studied with collagen I (C) and collagen IV (D) conjugated to the sensor and sRAGE in solution. Laminin and fibronectin could not be conjugated to the sensor.

Studies by others have indicated that expression of mRAGE on cultured cells augments cell affinity to surfaces coated with ECM proteins [24]. This interaction was demonstrated to be specific, although whether it was direct or mediated by adaptor or co-expressed proteins was never clarified. *In vitro* binding studies with blends of purified sRAGE and ECM proteins demonstrate that sRAGE binds with very high affinity to collagen I, collagen IV, and laminin. Mean dissociation constant (*K_D_*) values of 2.32 nM for collagen I, 3.67 nM for collagen IV, and 1.18 nM for laminin, were obtained for studies with sRAGE in the solid phase (Fig. 1*A–C*). Collagen I and IV affinities determined with sRAGE in the immobile phase were confirmed by reciprocal binding studies with sRAGE in the mobile phase, thus demonstrating specificity of the measured interaction. Laminin and fibronectin, however, could not be conjugated to the surface of the sensor. Importantly, no specific binding of sRAGE to fibronectin could be demonstrated (Fig. 1*D*). Table 2 summarizes the results.

**Table 2:** Kinetics of binding of extracellular matrix proteins in solution to solid-phase sRAGE.

Analyte	Concentration (nM)	*k_on_* (M^−1^ s^−1^)	*k_off_* (s^−1^)	*K_D_* (nM)
Collagen I	3.87	ND^a^	ND	ND
	19.40	5.18×10^4^	1.82×10^−4^	3.51
	38.70	3.50×10^4^	5.66×10^−5^	1.62
	58.10	1.43×10^4^	2.63×10^−5^	1.84
Collagen IV	6.94	ND	ND	ND
	34.70	2.44×10^4^	6.26×10^−5^	2.57
	69.40	2.15×10^4^	7.72×10^−5^	3.59
	104.00	1.67×10^4^	8.10×10^−5^	4.81
Laminin	11.10	7.54×10^4^	1.91×10^−4^	2.53
	22.20	1.06×10^5^	1.02×10^−4^	0.96
	33.30	1.36×10^5^	8.02×10^−5^	0.59
	44.40	1.71×10^5^	1.08×10^−4^	0.63
Fibronectin	6.25	ND	ND	ND
	12.50	ND	ND	ND
	18.75	ND	ND	ND
	25.00	ND	ND	ND

a Not determined due to undetectable specific binding.

### Determination of sRAGE organ biodistribution

**Figure 2:**
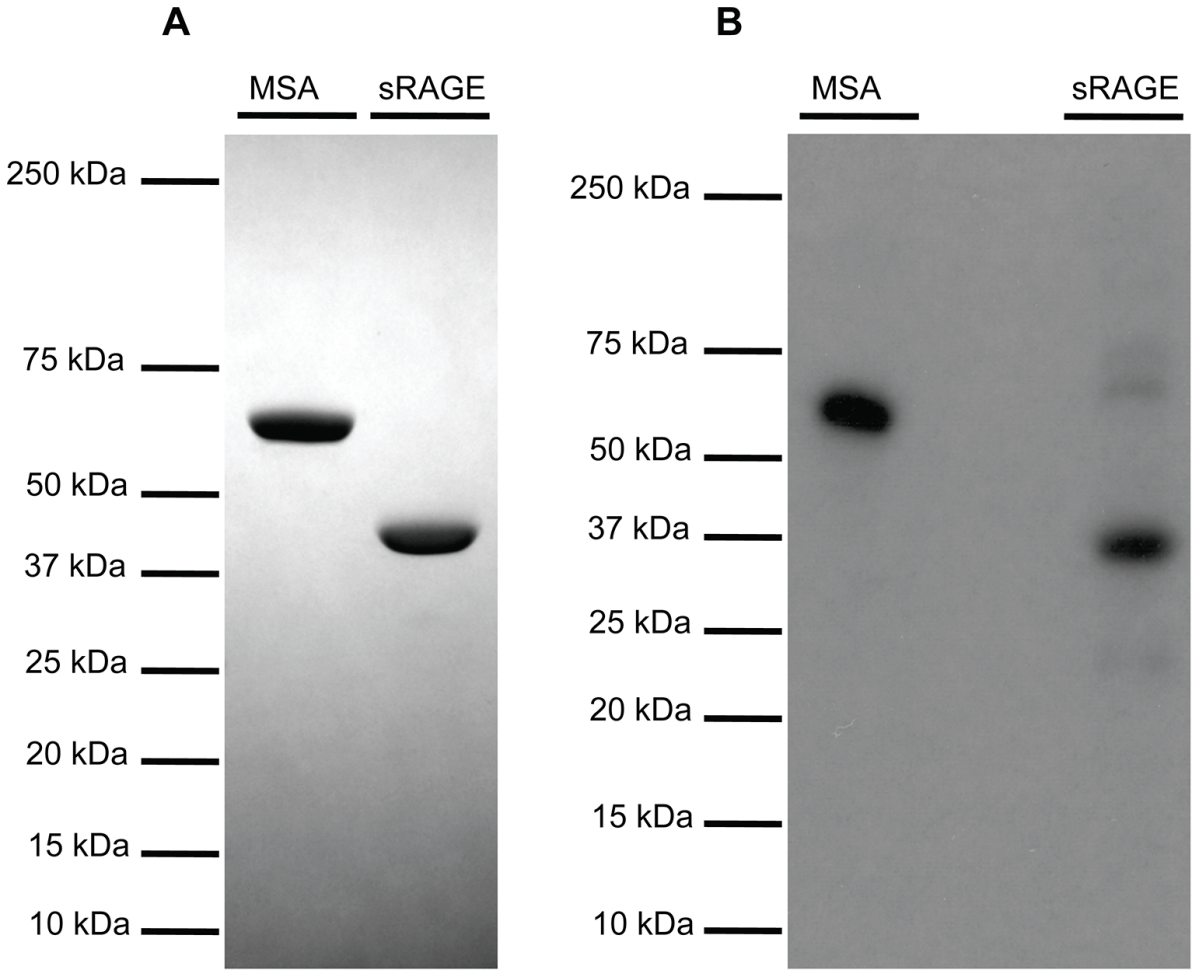
sRAGE and MSA used in biodistribution and clearance studies are very pure. (A) SDS-PAGE separation and Coomassie Brilliant Blue staining of ∼2.5 µg of MSA or mouse sRAGE preparations used for radiolabeling. (B) SDS-PAGE separation and gel autoradiography of ^125^I-labeled MSA and mouse sRAGE.

**Figure 3:**
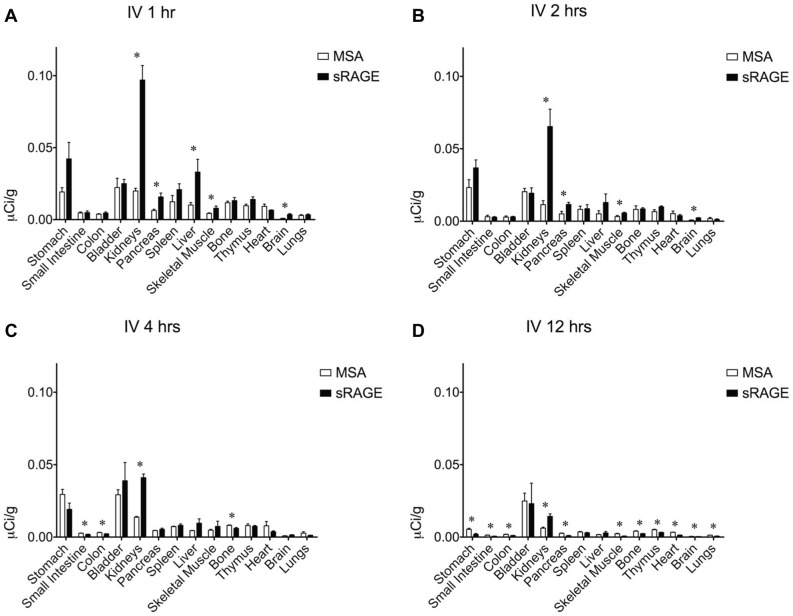
Organ biodistribution of intravenously-administered sRAGE or MSA in mice. Organ biodistribution of mouse sRAGE or MSA (A) 1 hour, (B) 2 hours, (C) 4 hours, and (D) 12 hours after intravenous injection of ∼1 µCi of tracer, corresponding to ∼0.6–1.4 µg of radiolabeled protein. Asterisks indicate statistically significant differences.

**Figure 4:**
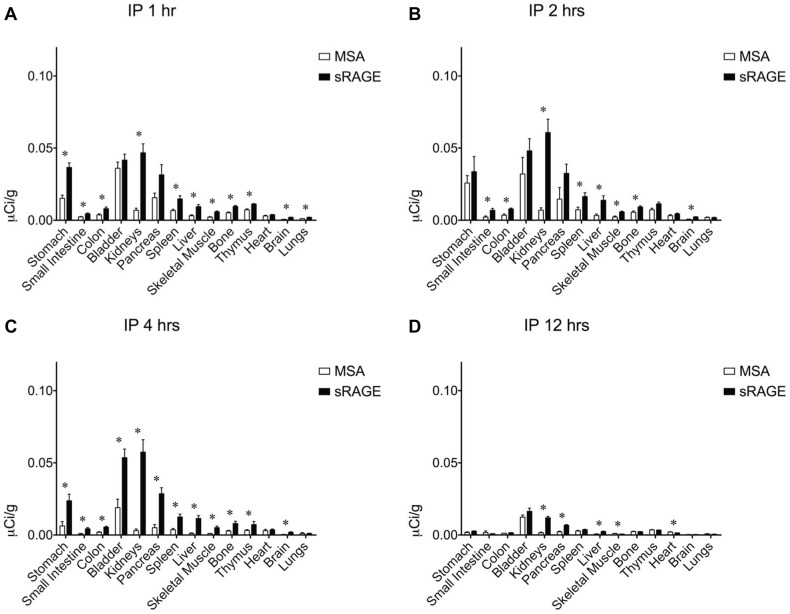
Organ biodistribution of intraperitoneally-administered sRAGE or MSA in mice. Organ biodistribution of mouse sRAGE or MSA (A) 1 hour, (B) 2 hours, (C) 4 hours, and (D) 12 hours after intraperitoneal injection of ∼1 µCi of tracer, corresponding to ∼0.6–1.4 µg of radiolabeled protein. Asterisks indicate statistically significant differences.

**Figure 5:**
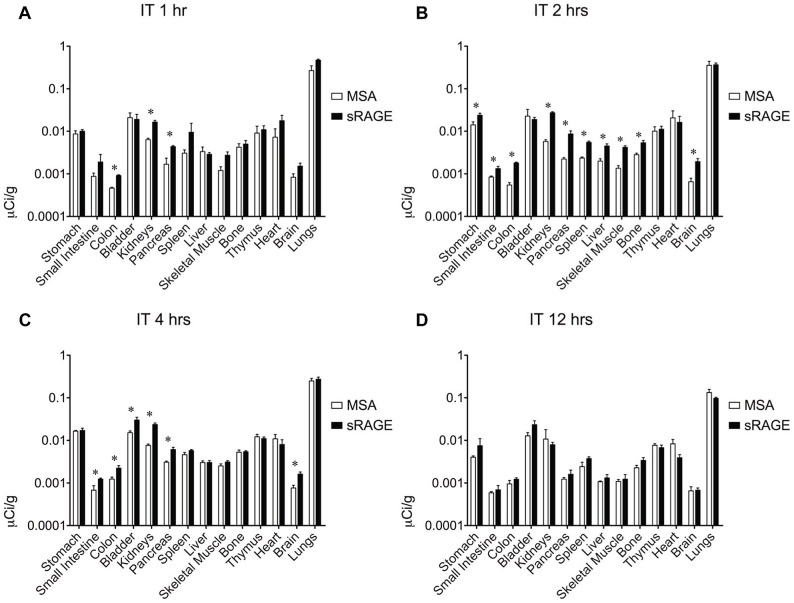
Organ biodistribution of intratracheally-administered sRAGE or MSA in mice. Organ biodistribution of mouse sRAGE or MSA (A) 1 hour, (B) 2 hours, (C) 4 hours, and (D) 12 hours after intratracheal instillation of ∼1 µCi of tracer, corresponding to ∼0.6–1.4 µg of radiolabeled protein. Asterisks indicate statistically significant differences.

To determine the most efficacious means of delivering sRAGE to the lung, clearance studies were performed with radiolabeled sRAGE or radiolabeled control MSA given via three common routes of administration. To confirm that the proteins being administered were adequately pure, SDS-PAGE and Coomassie Brilliant Blue gel staining were performed and indeed failed to detect the presence of proteins other than MSA (∼69 kDa) and sRAGE (∼42 kDa), which migrated as expected (Fig. 2*A*). Following radiolabeling, SDS-PAGE and gel autoradiography were performed to confirm ^125^I incorporation into sRAGE or MSA and removal of free iodine (Fig. 2*B*). Slight radiolabeled impurities are evident in both preparations but are unlikely to account for more than several percents of the total protein load. Small quantities of radiotracer were used so as to minimize any potential effect of these bioactive proteins on physiology. Intravenous (Fig. 3) and i.p. (Fig. 4) injections failed to deliver an appreciable quantity of sRAGE or MSA to the lung within the 12-hour window surveyed, by which time extensive renal clearance had already occurred. In contrast, i.t. instillation (Fig. 5) was very efficacious at delivering sRAGE and MSA to the lung, and was in fact the only feasible means of doing so.

### Characterization of sRAGE clearance from the lung and other organs

**Figure 6:**
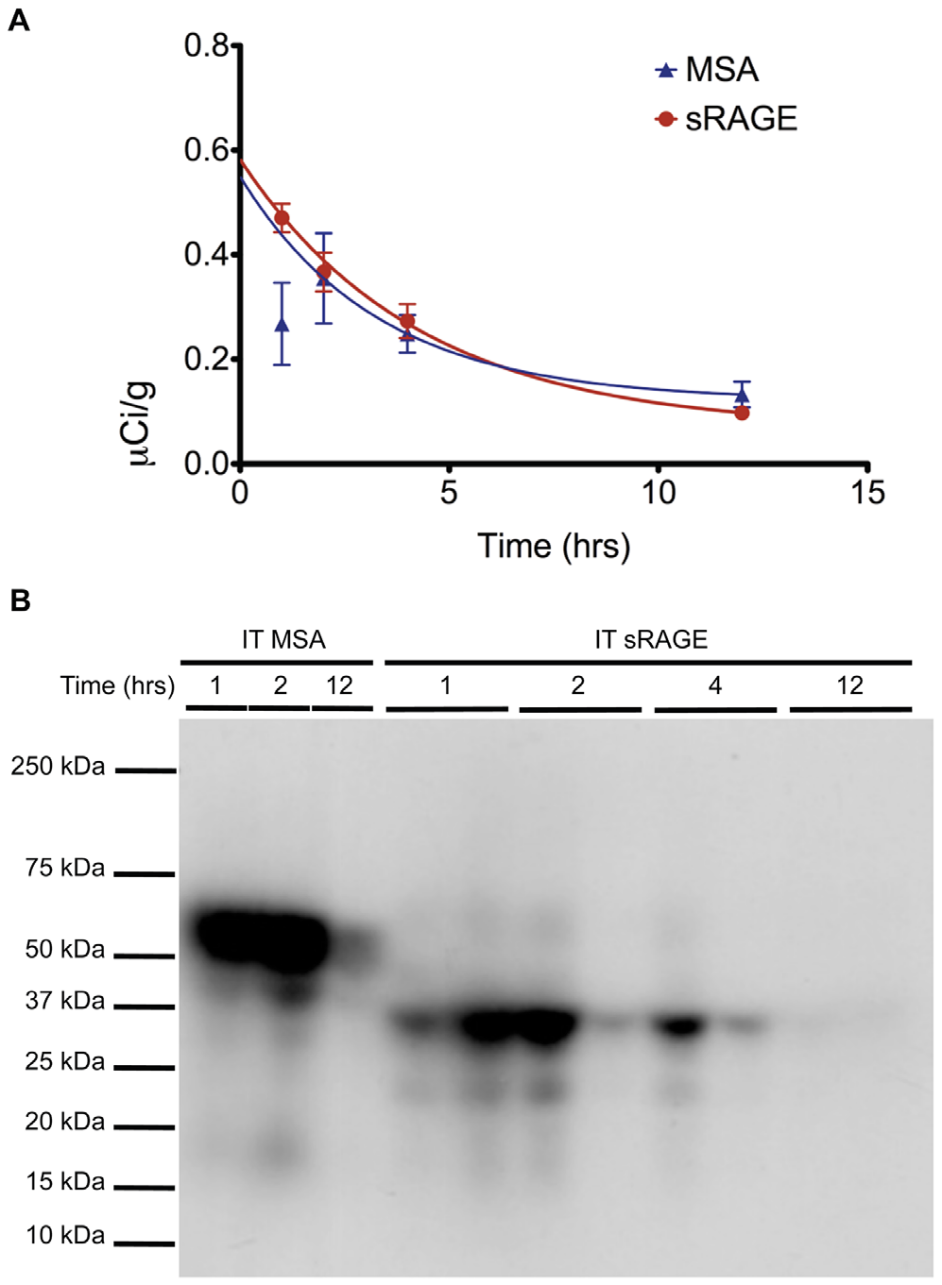
Clearance of intratracheally-administered sRAGE or MSA from mouse lung is rapid and non-proteolytic. (A) Exogenous mouse sRAGE or MSA persistence in mouse lung as a function of time. (B) SDS-PAGE separation and gel autoradiography of lung homogenates from mice administered i.t. mouse sRAGE or MSA and sacrificed at the indicated time points thereafter.

To investigate the kinetics of exogenous protein clearance the biodistribution data for intravenous, intraperitoneal, and intratracheal administration of ^125^I-labeled sRAGE (and ^125^I-labeled MSA, for the case of i.t. instillation) was plotted with respect to time, and mono-exponential decay fitting was performed. For i.v. administrations, data from the 1 hr time point onwards was used for fitting; for i.p. and i.t. administrations, where there is a lag time before the majority of the protein has translocated out of the peritoneal and pulmonary compartments, data from the 2 hr time point onwards was used for fitting. Bi-exponential and multi-exponential fittings were not taken into consideration because there is insufficient mechanistic understanding at the microscopic level of the processes by which MSA or sRAGE are cleared from lung and other organs. The half-lives of sRAGE organ elimination, determined from the above described nonlinear regression analyses, are summarized in Table 3. Both MSA and sRAGE (Fig. 6*A*, blue and red curves, respectively) were cleared rapidly from the lung, with half-lives of 2.29 hours for MSA and 2.98 hours for sRAGE. Catabolism is one of many possible mechanisms of protein clearance. To test whether degradative processes are involved in or compete with sRAGE clearance from lung, lung homogenates from animals that had received MSA or sRAGE by i.t. instillation were prepared, and proteins were separated by SDS-PAGE. Gel autoradiography was subsequently performed to detect the radiolabeled species present. Comparison with the gel autoradiograph of the ^125^I-labeled purified proteins (Fig. 2*B*) indicates that neither MSA nor sRAGE undergo appreciable proteolysis as they are eliminated from normal healthy lung (Fig. 6*B*).

**Table 3:** Organ elimination half-life of exogenous mouse sRAGE administered by three different routes.

Organ	*t_1/2_* (hrs, i.v.)	*t_1/2_* (hrs, i.p.)	*t_1/2_* (hrs, i.t.)
Stomach	2.92	5.04	2.98
Small intestine	1.28	2.82	ND^a^
Colon	1.58	3.46	ND
Bladder	ND	ND	ND
Kidneys	1.75	ND	ND
Pancreas	1.87	ND	3.90
Spleen	0.514	5.82	ND
Liver	0.591	ND	2.39
Skeletal muscle	ND	ND	3.74
Bone	1.65	ND	ND
Thymus	2.05	1.95	ND
Heart	2.13	8.95	1.27
Brain	1.78	ND	10.7
Lungs	0.359	1.74	2.98

a Not determined due to ambiguity, interruption, or lack of convergence of fit.

### Determination of exogenous sRAGE cellular localization in lung

**Figure 7:**
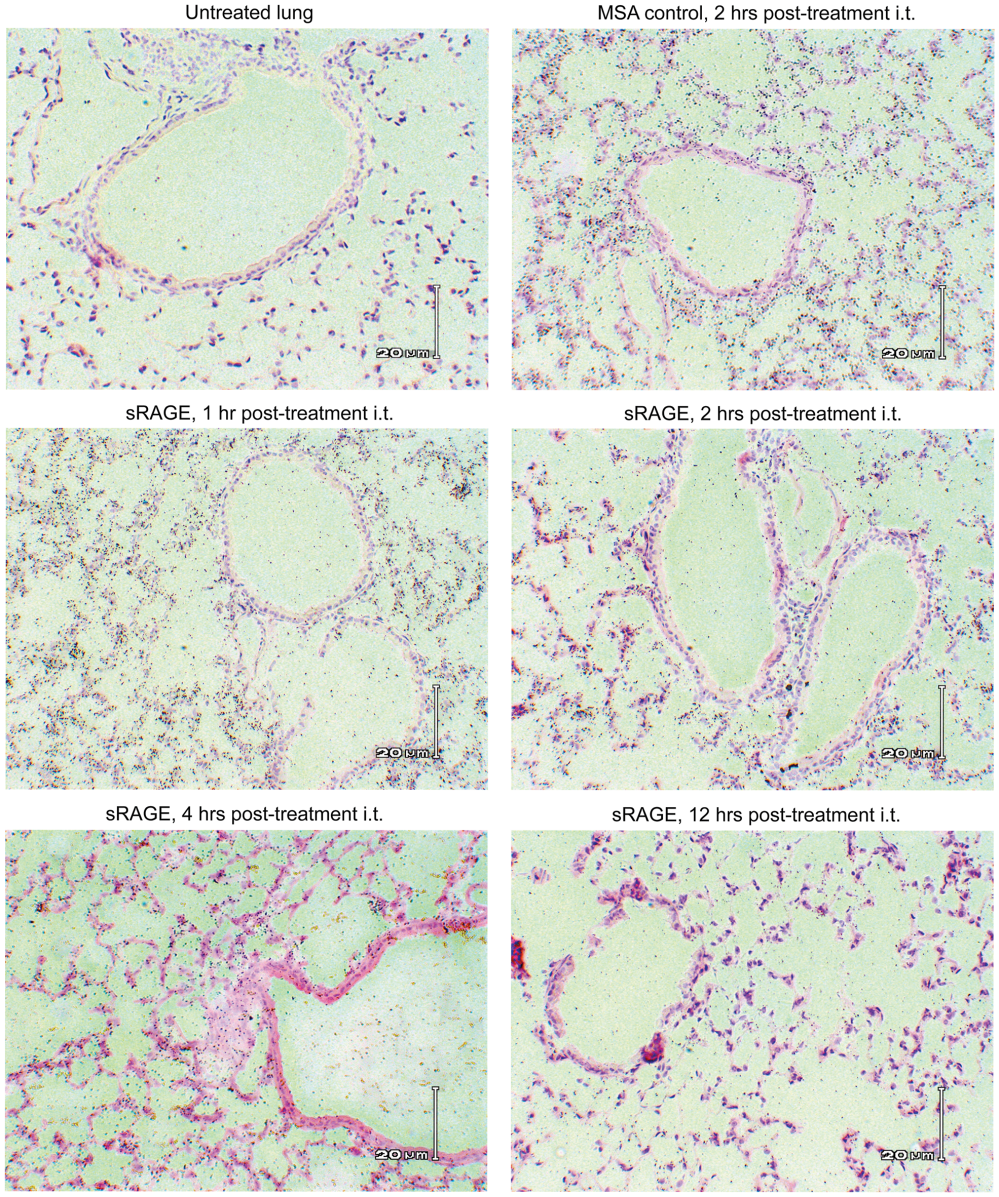
Clearance of intratracheally-administered sRAGE from mouse lung occurs at the alveolar-capillary interface. Representative light micrographs/autoradiographs recorded at 20× magnification of sections of lung from mice given mouse sRAGE (or MSA, shown for comparison) i.t. and sacrificed at the indicated time points thereafter. Lung from untreated mice serves as a control for background radiation.

To determine the pulmonary sites at which sRAGE egress occurs, autoradiography was performed on sections of lung from mice given radiolabeled sRAGE by i.t. instillation. Soluble RAGE and MSA rapidly reach the alveolar compartment and thence the circulation (Fig. 7). There is no apparent predilection of these proteins for the bronchial epithelium, type II alveolar epithelial cells, or alveolar macrophages.

## Discussion

Soluble RAGE has been widely utilized as a therapeutic agent in animal models of inflammatory disease. The mechanism of its anti-inflammatory action *in vitro* and *in vivo* is thought to be that of ligand sequestration away from cell surface receptors (including but not limited to membrane RAGE) that activate downstream inflammatory signaling cascades. The potential for sRAGE retention in ECM *in vivo*, as well as the existence of receptors of which sRAGE is a ligand, has not been adequately studied. Biolayer interferometry experiments performed on binary mixtures of sRAGE and individual ECM components demonstrate high-affinity reversible binding of sRAGE to collagen I and IV as well as to laminin, with no observable specific interaction between sRAGE and fibronectin (Fig. 1). The kinetics of this interaction are relatively slow, which may be a consequence of the random orientation of solid-phase conjugated sRAGE reducing the avidity to ECM proteins, and the formation of sRAGE oligomers in solution [39] competing with binding to solid-phase ECM proteins. This represents an advance from previous studies conducted using RAGE-expressing cell lines and coated ECM [24], for it demonstrates that RAGE-ECM protein interactions can occur directly and without mediation of bridging molecules or intracellular scaffolds that would modulate RAGE conformation and cell surface density. Furthermore, these results suggest that exogenous sRAGE delivered as a therapeutic may interact with exposed basement membrane components and thus compete for ECM binding sites with adhesion proteins expressed on overlying cells, thus modulating cellular adhesion and function.

Probing for cognate receptors of a ligand of interest may be accomplished by assessing biodistribution and clearance characteristics of the ligand as compared to those of a suitable control. MSA was used as a treatment control to distinguish between specific preferential organ biodistribution due to sRAGE binding partners and nonspecific preferential organ biodistribution due to greater vascular density or permeability. The biodistribution profile of sRAGE and MSA following i.p. (Fig. 3) or i.v. (Fig. 4) administration would seem to suggest preferential retention of sRAGE as compared to MSA in a number of organs, including kidneys, liver, spleen, stomach, small intestine, colon, pancreas, skeletal muscle, bone, and brain. However, when these two administration routes were compared to each other and to i.t. instillation (Fig. 5), no consistent pattern of organ biodistribution emerged, with the notable exception of sRAGE localization to the kidneys. Indeed, the observation of transient preferential sRAGE biodistribution to multiple organs (but with no consistency in this regard between the various administration routes tested) is unlikely to reflect sRAGE-binding sites in these organs, but rather to the more rapid kinetics of transport of sRAGE across intervening barriers. Soluble RAGE is approximately half the molecular weight of MSA, with a reported Stokes radius of 2.81 nm for human sRAGE [40] as compared to a Stokes radius of 3.55 nm for bovine serum albumin [41]. This has important implications for paracellular transport, for transport across the medium-sized (∼4–5.5 nm in diameter) and largest (∼25 nm in diameter) pores of the peritoneal membrane [42], as well as for transport across vascular basement membranes, the theoretical pore sizes of which may vary depending upon the tissue in question. Thus, with sRAGE traversing the diffusion barriers to the peripheral tissues more rapidly than does MSA, a transient apparent discrepancy in distribution between the two radiolabeled proteins arises. However, the preferential biodistribution of sRAGE to the kidneys is consistent and likely related to the size and charge properties of sRAGE and MSA. Soluble RAGE binds with high affinity to heparin and heparan sulfate, the latter of which is abundant in renal basement membrane. Moreover, sRAGE is sufficiently small to traverse the glomerular barrier during filtration (the effective glomerular pore size being ∼8 nm) and thus be excreted intact. In contrast, the negative charge and larger size of albumin restrict it from being filtered intact, and therefore this protein must be eliminated via other pathways.

Of three common routes of administration tested, sRAGE can be delivered to the pulmonary compartment solely via intratracheal instillation (Fig. 5). Other investigators have suggested that intraperitoneally-administered sRAGE has therapeutic benefit in mouse models of lung disease [43]. It is possible that in these cases, pulmonary injury facilitates sRAGE translocation from the circulation into the lung either through a regulated mechanism or damage to the pulmonary epithelium and vasculature. Finally, it is also possible that the low doses (hundreds of nanograms) of radiolabeled proteins given here fail to recruit low-affinity transport receptors in the pulmonary vasculature that gain relevance when much higher doses (dozens of micrograms) of sRAGE are given i.p. or i.v. These studies are of vital importance for investigators seeking to better understand RAGE's contribution to homeostasis and pathogenesis in diseases of the lung, the organ in which RAGE is expressed most significantly.

Exogenous sRAGE is rapidly cleared from the lung with clearance kinetics similar to that of MSA (Fig. 6*A*). Moreover, sRAGE remains intact in the lung (Fig. 6*B*) and effectively distributes to the alveoli, from whence it translocates into the circulation (Fig. 7). These results are surprising, as the lung is rich in many RAGE ligands, including heparin [34], heparan sulfate [44], collagen I and IV [45], and indeed RAGE itself [40] (membrane and soluble RAGE displays homo-oligomerization at low concentrations), all of which are also present in the alveolar basement membrane and pulmonary interstitium. This indicates that, despite the abundance of RAGE binding partners in the lung, in the healthy state these do not act as effective sites of sRAGE sequestration. It is important to note, however, that a number of previous studies have found an inverse relationship between molecular size and lung clearance rate of a variety of molecules [46], [47]. Therefore, as a significantly smaller molecule than albumin, sRAGE would be expected to demonstrate significantly faster clearance from lung. That sRAGE clearance is less rapid than MSA clearance may suggest retardation of sRAGE transport by transient interactions between sRAGE and its ligands. In this context it is also worth noting that an alveolar epithelial receptor, gp60, has been identified as a mediator of albumin uptake and transcytosis [48]. It is likely that such a designated system for return of protein into the circulation does not exist for sRAGE, which would thus have to rely on less rapid mechanisms of egress such as paracellular transport and fluid-phase endocytosis [49]. Regardless of the microscopic details of protein clearance from the lung, it is evident from these biodistribution and clearance studies that, should sRAGE be used as a therapeutic in pulmonary disease, it must be functionalized to greatly increase half-life or it must be administered frequently via the airways. These studies utilized an i.t. instillation approach of sRAGE administration, but they also suggest that intranasal and nebulized aerosol administration would be effective in delivering sRAGE to the pulmonary compartment.

## References

[1] Vlassara H, Brownlee M, Cerami A (1988) Specific macrophage receptor activity for advanced glycosylation end products inversely correlates with insulin levels in vivo. Diabetes 37: 456–461. 2837419 10.2337/diab.37.4.456

[2] Vlassara H, Moldawer L, Chan B (1989) Macrophage/monocyte receptor for nonenzymatically glycosylated protein is upregulated by cachectin/tumor necrosis factor. J Clin Invest 84: 1813–1820. 2556447 10.1172/JCI114366 PMC304059

[3] Wautier JL, Wautier MP, Schmidt AM, Anderson GM, Hori O, et al (1994) Advanced glycation end products (AGEs) on the surface of diabetic erythrocytes bind to the vessel wall via a specific receptor inducing oxidant stress in the vasculature: a link between surface-associated AGEs and diabetic complications. Proc Natl Acad Sci U S A 91: 7742–7746. 8052654 10.1073/pnas.91.16.7742 PMC44478

[4] Neeper M, Schmidt AM, Brett J, Yan SD, Wang F, et al (1992) Cloning and expression of a cell surface receptor for advanced glycosylation end products of proteins. J Biol Chem 267: 14998–15004. 1378843

[5] Pisetsky DS, Erlandsson-Harris H, Andersson U (2008) High-mobility group box protein 1 (HMGB1): an alarmin mediating the pathogenesis of rheumatic disease. Arthritis Res Ther 10: 209. 18598385 10.1186/ar2440 PMC2483460

[6] Klune JR, Dhupar R, Cardinal J, Billiar TR, Tsung A (2008) HMGB1: endogenous danger signaling. Mol Med 14: 476–484. 18431461 10.2119/2008-00034.Klune PMC2323334

[7] Du Yan S, Zhu H, Fu J, Yan SF, Roher A, et al (1997) Amyloid-beta peptide-receptor for advanced glycation endproduct interaction elicits neuronal expression of macrophage-colony stimulating factor: a proinflammatory pathway in Alzheimer disease. Proc Natl Acad Sci U S A 94: 5296–5301. 9144231 10.1073/pnas.94.10.5296 PMC24672

[8] Boyd JH, Kan B, Roberts H, Wang Y, Walley KR (2008) S100A8 and S100A9 mediate endotoxin-induced cardiomyocyte dysfunction via the receptor for advanced glycation end products. Circ Res 102: 1239–1246. 18403730 10.1161/CIRCRESAHA.107.167544

[9] Steiner J, Bogerts B, Schroeter ML, Bernstein HG (2011) S100B protein in neurodegenerative disorders. Clin Chem Lab Med 49: 409–424. 21303299 10.1515/CCLM.2011.083

[10] Volz HC, Laohachewin D, Seidel C, Lasitschka F, Keilbach K, et al (2012) S100A8/A9 aggravates post-ischemic heart failure through activation of RAGE-dependent NF-kappaB signaling. Basic Res Cardiol 107: 250. 22318783 10.1007/s00395-012-0250-z

[11] Frommhold D, Kamphues A, Dannenberg S, Buschmann K, Zablotskaya V, et al (2010) RAGE and ICAM-1 differentially control leukocyte recruitment during acute inflammation in a stimulus-dependent manner. BMC Immunol 12: 56. 10.1186/1471-2172-12-56 PMC3203087 21970746

[12] Brett J, Schmidt AM, Yan SD, Zou YS, Weidman E, et al (1993) Survey of the distribution of a newly characterized receptor for advanced glycation end products in tissues. Am J Pathol 143: 1699–1712. 8256857 PMC1887265

[13] Teismann P, Sathe K, Bierhaus A, Leng L, Martin HL, et al (2012) Receptor for advanced glycation endproducts (RAGE) deficiency protects against MPTP toxicity. Neurobiol Aging 33: 2478–2490. 22227007 10.1016/j.neurobiolaging.2011.12.006 PMC3712169

[14] Kataoka K, Ono T, Murata H, Morishita M, Yamamoto KI, et al (2012) S100A7 promotes the migration and invasion of osteosarcoma cells via the receptor for advanced glycation end products. Oncol Lett 3: 1149–1153. 22783409 10.3892/ol.2012.612 PMC3389638

[15] Miller AL, Sims GP, Brewah YA, Rebelatto MC, Kearley J, et al (2012) Opposing roles of membrane and soluble forms of the receptor for advanced glycation end products in primary respiratory syncytial virus infection. J Infect Dis 205: 1311–1320. 22262795 10.1093/infdis/jir826 PMC3308901

[16] Bierhaus A, Humpert PM, Morcos M, Wendt T, Chavakis T, et al (2005) Understanding RAGE, the receptor for advanced glycation end products. J Mol Med (Berl) 83: 876–886. 16133426 10.1007/s00109-005-0688-7

[17] Marsche G, Weigle B, Sattler W, Malle E (2007) Soluble RAGE blocks scavenger receptor CD36-mediated uptake of hypochlorite-modified low-density lipoprotein. FASEB J 21: 3075–3082. 17536039 10.1096/fj.07-8316com PMC4861206

[18] Chuong C, Katz J, Pauley KM, Bulosan M, Cha S (2009) RAGE expression and NF-kappaB activation attenuated by extracellular domain of RAGE in human salivary gland cell line. J Cell Physiol 221: 430–434. 19591173 10.1002/jcp.21873 PMC2914572

[19] Englert JM, Hanford LE, Kaminski N, Tobolewski JM, Tan RJ, et al (2008) A role for the receptor for advanced glycation end products in idiopathic pulmonary fibrosis. Am J Pathol 172: 583–591. 18245812 10.2353/ajpath.2008.070569 PMC2258251

[20] Cheng C, Tsuneyama K, Kominami R, Shinohara H, Sakurai S, et al (2005) Expression profiling of endogenous secretory receptor for advanced glycation end products in human organs. Mod Pathol 18: 1385–1396. 15933755 10.1038/modpathol.3800450

[21] Dahlin K, Mager EM, Allen L, Tigue Z, Goodglick L, et al (2004) Identification of genes differentially expressed in rat alveolar type I cells. Am J Respir Cell Mol Biol 31: 309–316. 15205179 10.1165/rcmb.2003-0423OC

[22] Fehrenbach H, Kasper M, Tschernig T, Shearman MS, Schuh D, et al (1998) Receptor for advanced glycation endproducts (RAGE) exhibits highly differential cellular and subcellular localisation in rat and human lung. Cell Mol Biol (Noisy-le-grand) 44: 1147–1157. 9846897

[23] Shirasawa M, Fujiwara N, Hirabayashi S, Ohno H, Iida J, et al (2004) Receptor for advanced glycation end-products is a marker of type I lung alveolar cells. Genes Cells 9: 165–174. 15009093 10.1111/j.1356-9597.2004.00712.x

[24] Demling N, Ehrhardt C, Kasper M, Laue M, Knels L, et al (2006) Promotion of cell adherence and spreading: a novel function of RAGE, the highly selective differentiation marker of human alveolar epithelial type I cells. Cell Tissue Res 323: 475–488. 16315007 10.1007/s00441-005-0069-0

[25] Goova MT, Li J, Kislinger T, Qu W, Lu Y, et al (2001) Blockade of receptor for advanced glycation end-products restores effective wound healing in diabetic mice. Am J Pathol 159: 513–525. 11485910 10.1016/S0002-9440(10)61723-3 PMC1850533

[26] Muhammad S, Barakat W, Stoyanov S, Murikinati S, Yang H, et al (2008) The HMGB1 receptor RAGE mediates ischemic brain damage. J Neurosci 28: 12023–12031. 19005067 10.1523/JNEUROSCI.2435-08.2008 PMC4597312

[27] Pullerits R, Brisslert M, Jonsson IM, Tarkowski A (2006) Soluble receptor for advanced glycation end products triggers a proinflammatory cytokine cascade via beta2 integrin Mac-1. Arthritis Rheum 54: 3898–3907. 17133598 10.1002/art.22217

[28] Schmidt AM, Yan SD, Wautier JL, Stern D (1999) Activation of receptor for advanced glycation end products: a mechanism for chronic vascular dysfunction in diabetic vasculopathy and atherosclerosis. Circ Res 84: 489–497. 10082470 10.1161/01.res.84.5.489

[29] Sourris KC, Morley AL, Koitka A, Samuel P, Coughlan MT, et al (2010) Receptor for AGEs (RAGE) blockade may exert its renoprotective effects in patients with diabetic nephropathy via induction of the angiotensin II type 2 (AT2) receptor. Diabetologia 53: 2442–2451. 20631980 10.1007/s00125-010-1837-2 PMC4926314

[30] Sternberg DI, Gowda R, Mehra D, Qu W, Weinberg A, et al (2008) Blockade of receptor for advanced glycation end product attenuates pulmonary reperfusion injury in mice. J Thorac Cardiovasc Surg 136: 1576–1585. 19114209 10.1016/j.jtcvs.2008.05.032

[31] Wendt T, Harja E, Bucciarelli L, Qu W, Lu Y, et al (2006) RAGE modulates vascular inflammation and atherosclerosis in a murine model of type 2 diabetes. Atherosclerosis 185: 70–77. 16076470 10.1016/j.atherosclerosis.2005.06.013

[32] Zeng S, Feirt N, Goldstein M, Guarrera J, Ippagunta N, et al (2004) Blockade of receptor for advanced glycation end product (RAGE) attenuates ischemia and reperfusion injury to the liver in mice. Hepatology 39: 422–432. 14767995 10.1002/hep.20045

[33] Ramasamy R, Yan SF, Schmidt AM (2009) RAGE: therapeutic target and biomarker of the inflammatory response–the evidence mounts. J Leukoc Biol 86: 505–512. 19477910 10.1189/jlb.0409230

[34] Englert JM, Ramsgaard L, Valnickova Z, Enghild JJ, Oury TD (2008) Large scale isolation and purification of soluble RAGE from lung tissue. Protein Expr Purif 61: 99–101. 18558495 10.1016/j.pep.2008.05.004 PMC2547992

[35] Hanford LE, Enghild JJ, Valnickova Z, Petersen SV, Schaefer LM, et al (2004) Purification and characterization of mouse soluble receptor for advanced glycation end products (sRAGE). J Biol Chem 279: 50019–50024. 15381690 10.1074/jbc.M409782200 PMC1868562

[36] Hofmann MA, Drury S, Fu C, Qu W, Taguchi A, et al (1999) RAGE mediates a novel proinflammatory axis: a central cell surface receptor for S100/calgranulin polypeptides. Cell 97: 889–901. 10399917 10.1016/s0092-8674(00)80801-6

[37] Abdiche Y, Malashock D, Pinkerton A, Pons J (2008) Determining kinetics and affinities of protein interactions using a parallel real-time label-free biosensor, the Octet. Anal Biochem 377: 209–217. 18405656 10.1016/j.ab.2008.03.035

[38] Do T, Ho F, Heidecker B, Witte K, Chang L, et al (2008) A rapid method for determining dynamic binding capacity of resins for the purification of proteins. Protein Expr Purif 60: 147–150. 18538581 10.1016/j.pep.2008.04.009

[39] Xie J, Burz DS, He W, Bronstein IB, Lednev I, et al (2007) Hexameric calgranulin C (S100A12) binds to the receptor for advanced glycated end products (RAGE) using symmetric hydrophobic target-binding patches. J Biol Chem 282: 4218–4231. 17158877 10.1074/jbc.M608888200

[40] Sarkany Z, Ikonen TP, Ferreira-da-Silva F, Saraiva MJ, Svergun D, et al (2011) Solution structure of the soluble receptor for advanced glycation end products (sRAGE). J Biol Chem 286: 37525–37534. 21865159 10.1074/jbc.M111.223438 PMC3199498

[41] Andrews P (1970) Estimation of molecular size and molecular weights of biological compounds by gel filtration. Methods Biochem Anal 18: 1–53. 4909316

[42] Rippe B (1993) A three-pore model of peritoneal transport. Perit Dial Int 13 Suppl 2: S35–38. 8399608

[43] Zhang H, Tasaka S, Shiraishi Y, Fukunaga K, Yamada W, et al (2008) Role of soluble receptor for advanced glycation end products on endotoxin-induced lung injury. Am J Respir Crit Care Med 178: 356–362. 18535257 10.1164/rccm.200707-1069OC

[44] Mizumoto S, Takahashi J, Sugahara K (2012) Receptor for advanced glycation end products (RAGE) functions as receptor for specific sulfated glycosaminoglycans, and anti-RAGE antibody or sulfated glycosaminoglycans delivered in vivo inhibit pulmonary metastasis of tumor cells. J Biol Chem 287: 18985–18994. 22493510 10.1074/jbc.M111.313437 PMC3365932

[45] Englert J (2009) A pathophysiologic evaluation of the receptor for advanced glycation end products (RAGE) in the lung [Ph.D.]. United States – Pennsylvania: University of Pittsburgh. 163 p.

[46] Hastings RH, Grady M, Sakuma T, Matthay MA (1992) Clearance of different-sized proteins from the alveolar space in humans and rabbits. J Appl Physiol 73: 1310–1316. 1447074 10.1152/jappl.1992.73.4.1310

[47] Effros RM, Mason GR (1983) Measurements of pulmonary epithelial permeability in vivo. Am Rev Respir Dis 127: S59–65. 6342486

[48] John TA, Vogel SM, Minshall RD, Ridge K, Tiruppathi C, et al (2001) Evidence for the role of alveolar epithelial gp60 in active transalveolar albumin transport in the rat lung. J Physiol 533: 547–559. 11389211 10.1111/j.1469-7793.2001.0547a.x PMC2278625

[49] Hastings RH, Folkesson HG, Matthay MA (2004) Mechanisms of alveolar protein clearance in the intact lung. Am J Physiol Lung Cell Mol Physiol 286: L679–689. 15003932 10.1152/ajplung.00205.2003

[50] Pace CN, Vajdos F, Fee L, Grimsley G, Gray T (1995) How to measure and predict the molar absorption coefficient of a protein. Protein Sci 4: 2411–2423. 8563639 10.1002/pro.5560041120 PMC2143013

